# Angiotensin II-Induced Long Non-Coding RNA *Alivec* Regulates Chondrogenesis in Vascular Smooth Muscle Cells

**DOI:** 10.3390/cells10102696

**Published:** 2021-10-09

**Authors:** Vishnu Amaram Samara, Sadhan Das, Marpadga A. Reddy, Vinay Singh Tanwar, Kenneth Stapleton, Amy Leung, Maryam Abdollahi, Rituparna Ganguly, Linda Lanting, Rama Natarajan

**Affiliations:** 1 Department of Diabetes Complications and Metabolism, Arthur Riggs Diabetes and Metabolism Research Institute, Duarte, CA 91010, USA; vsamara@mednet.ucla.edu (V.A.S.); sadhan.das1@cdri.res.in (S.D.); mreddy@coh.org (M.A.R.); vtanwar@coh.org (V.S.T.); kstapleton89@gmail.com (K.S.); amy.y.leung1@gmail.com (A.L.); mabdollahi@coh.org (M.A.); ganguly.rituparnaa@gmail.com (R.G.); llanting@coh.org (L.L.); 2 Irell and Manella Graduate School of Biological Sciences, Beckman Research Institute, City of Hope, Duarte, CA 91010, USA; 3 Division of Pharmacology, CSIR-Central Drug Research Institute, Lucknow, UP 226031, India

**Keywords:** Angiotensin II, lncRNAs, cardiovascular disease, vascular smooth muscle cells, chondrocytes, hypertension

## Abstract

Long non-coding RNAs (lncRNAs) play key roles in Angiotensin II (AngII) signaling but their role in chondrogenic transformation of vascular smooth muscle cells (VSMCs) is unknown. We describe a novel AngII-induced lncRNA *Alivec* (Angiotensin II-induced lncRNA in VSMCs eliciting chondrogenic phenotype) implicated in VSMC chondrogenesis. In rat VSMCs, *Alivec* and the nearby gene *Acan*, a chondrogenic marker, were induced by growth factors AngII and PDGF and the inflammatory cytokine TNF-α. AngII co-regulated *Alivec* and *Acan* through the activation of AngII type1 receptor signaling and Sox9, a master transcriptional regulator of chondrogenesis. *Alivec* knockdown with GapmeR antisense-oligonucleotides attenuated the expression of AngII-induced chondrogenic marker genes, including *Acan*, and inhibited the chondrogenic phenotype of VSMCs. Conversely, *Alivec* overexpression upregulated these genes and promoted chondrogenic transformation. RNA-pulldown coupled to mass-spectrometry identified Tropomyosin-3-alpha and hnRNPA2B1 proteins as *Alivec*-binding proteins in VSMCs. Furthermore, male rats with AngII-driven hypertension showed increased aortic expression of *Alivec* and *Acan*. A putative human ortholog *ALIVEC*, was induced by AngII in human VSMCs, and this locus was found to harbor the quantitative trait loci affecting blood pressure. Together, these findings suggest that AngII-regulated lncRNA *Alivec* functions, at least in part, to mediate the AngII-induced chondrogenic transformation of VSMCs implicated in vascular dysfunction and hypertension.

## 1. Introduction

Cardiovascular diseases (CVDs), such as hypertension and atherosclerosis, are leading causes of morbidity and mortality worldwide [1]. Vascular smooth muscle cells (VSMCs) in the arterial wall maintain vascular tone and blood pressure and are under the control of the renin–angiotensin system (RAS)-Angiotensin II (AngII) system. AngII, the primary effector of the RAS pathway, is a potent vasoconstrictor and regulator of blood pressure. Dysregulation of RAS or abnormal AngII signaling is implicated in hypertension and atherosclerosis [2].

In CVD or vascular injury, dysregulated growth factor and AngII signaling promotes VSMCs to switch from a contractile to synthetic phenotype [3]. The synthetic phenotype manifests in increased VSMC proliferation, hypertrophy, migration, inflammation and the key processes associated with the pathogenesis of arterial stenosis/restenosis, hypertension and atherosclerosis [4,5,6,7]. Furthermore, the synthetic VSMCs tend to transform into chondrocyte-like cells, which promotes extracellular calcium deposition and vascular dysfunction associated with these pathologies [8,9,10]. Aggrecan (Acan) is an extracellular matrix protein that is prominent in chondrocytes during cartilage formation and is upregulated in aortic VSMCs after injury [10]. The transcription factor (TF) Sox9, which regulates chondrogenesis, is associated with VSMC synthetic/chondrocyte phenotype and promotes extra-cellular matrix (ECM) alterations and calcium deposition [11]. However, the mechanisms involved in AngII-mediated phenotypic transformation of VSMC to chondrocyte-like cells are not well understood. 

Long non-coding RNAs (lncRNAs) are a group of non-coding RNAs (ncRNAs) that are more than 200 nucleotides in size and are processed like protein coding mRNAs but lack protein-coding potential [12]. LncRNAs have diverse functions and regulate gene expression at the level of transcription through the interaction with and recruitment of TFs, chromatin modifier proteins and ribonucleoproteins to specific target gene loci, or via the post-transcriptional regulation of microRNAs and signaling proteins [13]. Genome-wide association studies (GWAS) identified several single nucleotide polymorphisms (SNPs) associated with CVDs that reside in the lncRNA loci [14]. LncRNAs regulate various physiological and pathological processes [15]. In VSMCs they regulate cell proliferation, migration, reactive oxygen species (ROS) production and inflammation, key factors associated with CVDs [16,17]. We identified the first lncRNAs regulated by AngII in rat VSMCs (RVSMCs) using integrated analysis of RNA-seq data with ChIP-seq datasets from histone H3K4me3 and H3K36me3 profiling [18]. Since then, several VSMC lncRNAs such as *SENCR*, *MYOSLID* and *SMILR* were described and found to play key roles in CVDs [19,20,21]. Another abundant nuclear lncRNA, *NEAT1*, was reported to be involved in VSMC phenotypic switching [22]. We also reported that the AngII-induced lncRNA *Giver* regulated oxidative stress, inflammation and proliferation in VSMCs through epigenetic mechanisms. *Giver* was upregulated in aortas of AngII treated hypertensive mice and in individuals with hypertension [23]. Furthermore, we found that lncRNA interactions with enhancers had functional roles in AngII-induced gene expression in RVSMCs [24]. 

Herein, we identified another novel AngII-induced lncRNA and characterized its regulation and functional role in RVSMCs. We named this lncRNA *Alivec* (AngII-induced lncRNA in vascular smooth muscle cells eliciting chondrogenic phenotype). In RVSMCs, lncRNA *Alivec* and its nearby chondrogenic marker gene *Acan* were highly upregulated by AngII, a process mediated through the AngII type 1 receptor (AT1R) and Sox9, a master regulator of chondrogenesis. Functional studies indicated that *Alivec* regulated the AngII-induced expression of *Acan* and other genes associated with chondrogenesis. In addition, we found that *Alivec* interacted with the contractile protein tropomyosin-3-alpha (Tpm3) and the RNA-binding protein hnRNPA2B1. *Alivec* and *Acan* were upregulated in aortas from rats with AngII-induced hypertension. Interestingly, the analysis of a putative human *ALIVEC* locus revealed multiple quantitative trait loci (QTLs) that are potentially associated with CVD, and human VSMCs treated with AngII showed upregulation of the human ortholog. These findings indicate that the novel AngII-induced lncRNA *Alivec* drives phenotypic switching of contractile VSMCs to a chondrogenic phenotype, associated with hypertension.

## 2. Materials and Methods

### 2.1. Animal Studies

All animal studies were conducted in accordance with protocols approved by the Institutional Animal Care and Use Committee (IACUC) of the Beckman Research Institute of City of Hope. (IACUC approval number is 14002).

### 2.2. Cell Culture and Treatment

RVSMCs were isolated from de-endothelialized thoracic aortas of 12-week-old male Sprague–Dawley rats (Charles River Labs, Wilmington, MA, USA) by enzymatic digestion after removal of endothelial layers, as described [23]. Cells were cultured in M199 medium supplemented with 10% fetal bovine serum (FBS), 1% penicillin/streptomycin antibiotics and 2.5 µg/mL plasmocin. Human VSMCs (HVSMCs) were purchased from American Type Culture Collection (ATCC, Manassas, VA, USA) (PCS-100-012^TM^) and cultured in M231 medium with smooth muscle growth supplement (Gibco, Waltham, MA, USA). For all the experiments, RVSMCs and HVSMCs between passages 3 and 6 were used. For in vitro experiments, the complete medium was replaced with serum-free medium containing 0.2% BSA for 24 h prior to stimulation with AngII (100 nM, Bachem, Torrance, CA, USA), platelet-derived growth factor-BB (PDGF-BB, 10 ng/mL), or tumor necrosis factor-alpha TNF-α (50 ng/mL, 210-TA, R&D Systems, Minneapolis, MN, USA).

### 2.3. Treatment with Inhibitors of the AT1R and Signal Transduction Pathways

RVSMCs were treated with the signaling and pathway-specific inhibitors as described [24,25]. Briefly, RVSMCs were pre-treated with inhibitors of p38 MAP kinase (SB202190, 5 µM, Cell Signaling Technology, Danvers, MA, USA), Phospho-p42/p44 MAPK or Erk1/2 (U0126,10 µM Cell Signaling Technology), Src (PP1, 10 µM, Calbiochem, Billerica, MA, USA), JAK (Inhibitor I, 10 µM), the AT1R antagonist losartan (10 µM, Merck, Whitehouse Station, NJ, USA) or the vehicle DMSO for 1 h prior to treatment with AngII (100 nM, 3 h).

### 2.4. RNA Isolation and RT-qPCR

Total RNA was isolated from the rat aortas, RVSMCs and HVSMCs, using TRIzol and an RNeasy mini kit (Qiagen, Germantown, MD, USA). cDNA was synthesized using a high-capacity cDNA reverse transcription kit (Thermo Fisher Scientific, Carlsbad, CA, USA). RT-qPCR was performed using SYBR green master mix (Applied Biosystems, Foster City, CA, USA) and analyzed on a 7500 Fast Real Time PCR system (Thermo Fisher Scientific). Relative gene expression was analyzed using the 2 ^−ΔΔ^Ct method and normalized to *Ppia* (rat) and *GAPDH* (human), as described [23,24]. Sequences of primers used are listed in Supplementary Table S1.

### 2.5. Cellular Fractionation

Cytoplasmic and nuclear fractions from RVSMCs were purified and RNA isolated using a cellular fractionation kit (Norgen, ON, Canada). Briefly, RVSMCs were incubated on ice with cold lysis solution and centrifuged to separate cytoplasmic and nuclear components [23]. RNA was isolated from each fraction followed by RT-qPCR, as described above.

### 2.6. RNA Fluorescence In Situ Hybridization (RNA-FISH)

RNA–FISH was performed to determine the subcellular localization of *Alivec* using a ViewRNA ISH Cell Assay Kit (Affymetrix, Santa Clara, CA, USA), as described [23]. Branched DNA signal amplification probes manufactured by Affymetrix eBiosciences (Thermo Fisher Scientific) were used to target *Alivec*. RVSMCs were plated in 4-chamber slides (LAB-TEK Nunc, Rochester, NY, USA) in M199 complete medium, treated with AngII (100 nM) for 3 h and fixed with 4% formaldehyde, with probes and signal amplification reagents added. Cells stained with *Ppia* probes served as a positive control and cells without a probe served as a negative control. Images were captured with a Zeiss Observer Z1 wide-field microscope and processed using ZEN Blue software (Zeiss, Oberkochen, Germany) with settings kept constant across all the samples.

### 2.7. 5′ and 3′ Rapid Amplification of cDNA Ends (RACE)

To define the specific ends of *Alivec* transcript, 5′ and 3′ RACE-PCRs were performed using a FirstChoice RLM-RACE kit (Thermo Fisher Scientific). The PCR products were Sanger sequenced and, using SnapGene software (GSL Biotech, Chicago, IL, USA), the sequences aligned to the *Alivec* genomic locus to define the ends. The coding potential of the full length *Alivec* sequence was determined using the coding potential calculator tool web-based portal version 2.0 (CPC2) [26].

### 2.8. In Vitro Transcription and Translation

The complete *Alivec* cDNA sequence (Supplementary Table S2) was synthesized and cloned into pcDNA3.1+ vector (Thermo Fisher Scientific) by a commercial vendor (Vector Builder Inc, Chicago, IL, USA) to generate a pcDNA3.1-*Alivec* construct. Then, the linearized pcDNA3.1-*Alivec* construct was subjected to in vitro transcription and translation assays to verify the coding potential of *Alivec* using the T7 TNT quick coupled transcription/translation system (Promega, Madison, WI, USA). Control pcDNA3.1 plasmid with luciferase expressing from the T7 promoter was used as a positive control and no plasmid template (Thermo Fisher Scientific) was used as a negative control. The translation products from these reactions were loaded on to SDS-PAGE gel and then transferred onto a positively charged nylon membrane. Protein products were detected with a streptavidin antibody and western blue reagent (Promega).

### 2.9. Transient Transfection of RVSMCs with Plasmids, GapmeRs and siRNAs

RVSMCs were transiently transfected with antisense-locked nucleic acid (LNA)-modified GapmeRs (100 nM), targeting *Alivec* (*Alivec*Gap) or a non-targeting control (NCGap) obtained from Qiagen, and small interfering RNAs (siRNAs, 10 nM) targeting Sox9 or the control non-targeting siRNAs (Horizon, Lafayette, CO, USA) using Lipofectamine RNAiMax (Thermo Fisher Scientific), as described [23]. Transfected cells were serum depleted for 24 h prior to the AngII treatment (100 nM, 3 h) and RNA was collected 48–72 h after transfection. RVSMCs were transiently transfected with expression plasmids for *Alivec* and pcDNA-Sox9 (a kind gift from Maike Sander, UCSD, San Diego, CA, USA) using Lipofectamine 3000 transfection reagent (Thermo Fisher Scientific). Sequences of siRNAs and GapmeRs used in this study are listed in Supplementary Table S3.

### 2.10. Affymetrix Gene Array Analyses 

Microarray hybridization, data acquisition and the initial analysis were performed by the Integrative Genomics Core of City of Hope. Biotinylated cDNA derived from total RNA was hybridized with the Clariom S Assay GeneChip array for rat transcriptome wide gene expression profiling (Thermo Fisher Scientific). Three independent replicates were performed for each group of samples. Raw intensity data in CEL file format were imported into the Partek Genomics Suite (version 6.6, Partek Inc., St. Louis, MO, USA) and preprocessed and normalized using the Robust Multichip Average method. The probe sets with no or low expression (normalized log2 signal intensity less than 6) were removed from further analysis. Comparisons between NCGap and *Alivec*Gap transfected at the basal level and AngII-treated RVSMCs were performed using the analysis of variance method in Partek. Statistically significant differentially expressed genes (DEG) were defined using the criteria of absolute fold change ≥1.20 and a *p*-value of <0.05. Biological functions and network analysis of DEG was carried out with TOPPGENE, DAVID and KEGG pathway tools. Transcription Factor Affinity Prediction (TRAP) web tools were used for analyzing the TF-binding motifs.

### 2.11. Identification of Rat lncRNA Alivec

Publicly available RNA-seq data (GSE38056) and ChIP-seq data (GSE95067), previously published by our laboratory [18,24], were used to identify lncRNA *Alivec* and the enrichment of H3K27ac overlapping *Alivec* locus in rat VSMCs. The RNAseq data from rat VSMCs treated ± AngII for 3 h were aligned to rat genome assembly rn4 (Baylor 3.4/rn4) with spliced transcript alignment to a reference (STAR, version 2.6.0.a) aligner tool using default parameters. Integrative Genomics Viewer was used to visualize the RNA-seq and ChIP-seq datasets.

### 2.12. Alcian Blue Staining to Determine Chondrogenic Phenotype 

Following knockdown and the overexpression of *Alivec*, RVSMCs were incubated overnight with 0.1% alcian blue (Sigma-Aldrich, Burlington, MA, USA) in 0.1 M HCl. Cells were washed, bound and stain extracted with 6 M guanidinium hydrochloride for 8 h, with the absorbance read at 620 nm [27].

### 2.13. AngII-Infused Rat Model of Hypertension and Vasculopathy

Osmotic minipumps (Alzet model 2002, Cupertino, CA, USA) filled with AngII or vehicles were implanted subcutaneously in 12-week-old male Sprague–Dawley rats (3 rats/group). AngII was delivered at a rate of 200 ng/kg/min for 28 days [28]. During the final week of the experiment, blood pressure was measured using a tail cuff system (Visitech, Apex, NC, USA). At the end of the experiment, rats were humanely euthanized by CO_2_ and aortas harvested for RNA isolation and immunohistochemistry.

### 2.14. Tissue Staining and Immunohistochemistry

Aortas from AngII- and PBS-infused rats were fixed in 10% formalin, dehydrated using a series of alcohol levels (70%, 80%, 90%, and 100%), embedded in paraffin and sectioned (5 µm thickness) with a microtome. Sections were rehydrated and boiled in retrieval solution (Tris pH 6.0), cooled to room temperature for 20 min and placed in Tris-buffer saline-Tween (TBST). The slides were then incubated with a peroxidase block solution (3% H_2_O_2_). Non-specific binding was prevented by incubation in a blocking reagent (10% normal goat serum) for 20 min. Slides were then incubated with primary antibodies overnight at 4 °C. The primary antibodies used were Anti-alpha smooth muscle actin (alpha-SMA, Abcam, 1:1000 dilution), anti-transgelin (SM22), Proteintech, rabbit polyclonal, 1:50 dilution), anti-Runx1 (Proteintech, rabbit polyclonal, 1:1000 dilution) and anti-Aggrecan (Acan, Proteintech, rabbit polyclonal, 1:800 dilution) (Supplementary Table S4). The slides were washed 3 times in TBST and incubated with a secondary antibody (Vector Laboratories, 1:200) for 1 h at room temperature. The slides were washed 3 times in TBST and incubated with Vectastain ABC reagent (Vector Laboratories, Burlingame, CA, USA) for 30 min. To develop the color, the slides were incubated with 3, 3′-diaminobenzidine (DAB) substrates for 1–2 min. The slides were then counterstained with hematoxylin and mounted with coverslips. All slides were examined by light microscopy (X200) (Keyence, Osaka, Japan). 

### 2.15. Alivec RNA-Pulldown and Mass Spectrometry

*Alivec* RNA-pulldown assays were performed with lysates from RVSMCs treated with AngII, using published methods with some modifications [29]. The *Alivec*-expressing plasmid, pcDNA-Alivec, was used as a template in an in vitro transcription kit (Roche) to generate *Alivec* RNA. *Alivec* RNA or polyA RNA (the negative control) were biotin-labeled using an RNA 3′ Desthiobiotinylation Kit (Thermo Fisher Scientific). RNA-pulldown assays were performed using the Pierce Magnetic RNA–protein pulldown kit (Thermo Fisher Scientific) following the manufacturer’s protocol. Briefly, protein lysates (100 µg) from RVSMCs, treated with AngII (100 ng/mL, 3 h), were incubated with biotin-labeled *Alivec* or polyA RNA probes (100 pmol) and yeast tRNA (30 µg) at 4 °C for 2 h. The bound RNA–protein complexes were incubated with streptavidin beads for 2 more hours. The complexes were washed 5 times to remove non-specific binding proteins. Proteins were eluted using TRIS buffer and subjected to mass spectrometry (MS) analysis at the City of Hope Proteomics Core. The scaffold tool (Proteome Software Inc, Portland, OR, USA) was used to identify and validate the MS/MS-based peptides. Protein identifications were accepted if they contained at least 2 identified peptides and could be established with a minimum of 99.0% probability with the Scaffold local FDR algorithm. For validation of mass spectrometry results, eluted proteins were analyzed by Western blotting with antibodies against hnRNPA2B1 (1:1000) (Origene, Rockville, MD, USA) and Tpm3 (1:1000) (Genetex, Irvine, CA, USA) (ST III). 

### 2.16. UV-RNA Immunoprecipitation (RIP) Assay

The assay was performed as described [30]. Briefly, 1.0 × 10^7^ RVSMCs treated with AngII for 3 h were cross-linked with UV light using Stratalinker (1200 µjoules/cm^2^) and lysed with lysis buffer. The lysates were diluted in RIP buffer and incubated with 5 µg each of anti-Tpm3 (Genetex) or rabbit IgG as the controls. The antibody-bound RNA–protein complexes were captured on magnetic protein G beads and bound RNA was isolated, followed by an RT-qPCR analysis. 

### 2.17. Data Deposition

Affymetrix data are deposited in the Gene Expression Omnibus (accession number: GSE183857).

### 2.18. Statistical Analysis

All experiments were performed at least 3 times unless otherwise mentioned in the figure legend. Data were analyzed using GraphPad PRISM 8 (GraphPad, San Diego, CA, USA). The data were represented as the mean ± standard deviation (SD). A *p*-value <0.05 was considered statistically significant based on unpaired two-tailed *t*-tests for two groups and one-way ANOVA with Dunnett’s or Tukey’s multiple comparison tests for multiple groups. Normal data distributions were confirmed using the Shapiro–Wilk normality test.

## 3. Results

### 3.1. Alivec Is an AngII-Induced lncRNA Adjacent to Chondrogenic Gene Acan in RVSMCs

**Figure 1:**
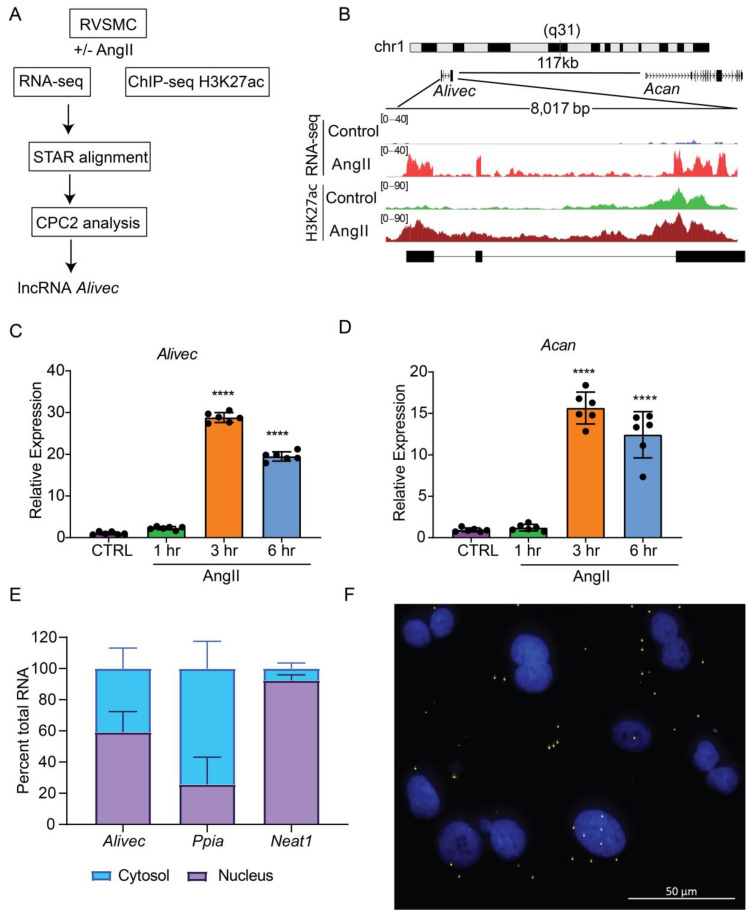
*Alivec* is an AngII-induced enhancer-associated lncRNA adjacent to chondrogenic gene *Acan* in RVSMCs. (**A**) Schematic diagram depicting RNA-seq and H3K27ac ChIP-seq alignment pipeline for the identification of lncRNA *Alivec* (AngII-induced lncRNA in vascular smooth muscle cells eliciting chondrogenic phenotype) exons, overlapping H3 lysine 27 acetylation (H3K27ac) enrichment and *Alivec*’s coding potential, which was determined using the software CPC2 (coding potential calculator 2). (**B**) Schematic showing genomic organization of *Alivec* and the neighboring gene *Acan* in the rat genome. Integrative Genomics Viewer (IGV) tracks showing *Alivec* locus with representative RNA-seq tracks (RNA-Seq) and H3K27ac ChIP-seq tracks (H3K27ac) from control- and AngII-treated RVSMCs. (**C**,**D**) RT-qPCR analysis of *Alivec* and *Acan* expression in RVSMCs treated ± AngII (100 nM) for the indicated time periods. Data presented as mean ± SD, *n* = 6 biological replicates, one-way ANOVA followed by Dunnett’s multiple comparisons test and **** *p* < 0.0001 vs. control untreated cells (CTRL (**E**) RT-qPCR analysis of *Alivec*, *Ppia* and *Neat1* showing their relative enrichment in cytosolic and nuclear fractions of AngII-treated RVSMCs. (**F**) Subcellular localization of *Alivec* in AngII-treated RVSMCs, determined by RNA–FISH analysis. *Alivec* is shown as distinct yellow spots and nuclei are stained with DAPI (blue). Scale bar 50 µm.

We analyzed RNA-seq data previously generated in our laboratory from RVSMCs treated with AngII (100 nM, 3 h) [18] using STAR aligner and observed that a previously identified novel lncRNA (*lnc Ang26*), which we named *Alivec*, was highly induced by AngII (Figure 1A). To further characterize the *Alivec* locus, we integrated the RNA-seq data with histone H3K27ac (enhancer mark) ChIP-seq data from AngII treated RVSMCs [24]. Combined RNA-seq and ChIP-seq data showed that the lncRNA *Alivec* locus overlaps with an AngII-induced H3K27ac enriched region (Figure 1B). *Alivec* has 3 exons and the gene is located on rat chromosome 1 adjacent (117 kb distance) to the protein-coding gene *Acan* (Figure 1B). RNA-seq analyses also showed that the expression of the nearby gene *Acan*, was likewise increased by AngII. Furthermore, RT-qPCR validation showed that RVSMCs exposed to AngII displayed marked induction of *Alivec* expression (up to 30-fold) within 3 h of treatment; this persisted even at 6 h compared to the control cells (Figure 1C). Under the same conditions, the induction of *Acan* was also observed (Figure 1D), suggesting a potential role for *Alivec* in the regulation of *Acan* expression by AngII. This was interesting, as *Acan* codes for the protein aggrecan, which is known to be induced by growth factors and cytokines and is also a key biomarker of chondrogenesis associated with VSMC dysfunction in CVDs [31]. 

Next, we performed experiments to further characterize *Alivec*. Rapid amplification of cDNA end (RACE)-PCR experiments verified the 5′ and 3′ ends of *Alivec* and defined the total transcript size to be 2275 nucleotides (Supplementary Figure S1A,B and Supplementary Table S2). Considering the localization of lncRNAs in the nucleus or cytoplasm can determine their functions, [32] we examined the cellular localization of lncRNA *Alivec*. In AngII-treated RVSMCs, sub-cellular fractionation followed by RT-qPCR showed that *Alivec* is distributed in the nucleus and cytosol (Figure 1E). *Ppia* and a lncRNA *Neat1* served as controls for cytoplasmic and nuclear fractions, respectively (Figure 1E). RNA–FISH experiments with branched DNA probes, further confirming nuclear and cytoplasmic localization of *Alivec*, as indicated by the presence of distinct spots/foci distributed in both compartments (Figure 1F). These spots were not visible in the absence of the probes (Supplementary Figure S1C). The protein-coding potential analysis of *Alivec* (coding potential calculator version 2.0, CPC2) showed that it had a coding probability of 0.31, classifying it as a non-coding transcript. The lack of coding potential was confirmed by in vitro transcription/translation assays using pcDNA *Alivec* plasmids, which showed no detectable peptide product from *Alivec*, as compared to the positive luciferase control (Supplementary Figure S1D,E). Together, these results indicate that *Alivec* is an AngII-induced lncRNA in RVSMCs. 

### 3.2. AngII-induces Alivec and Acan Expression via Activation of AT1R and Src Kinase

**Figure 2:**
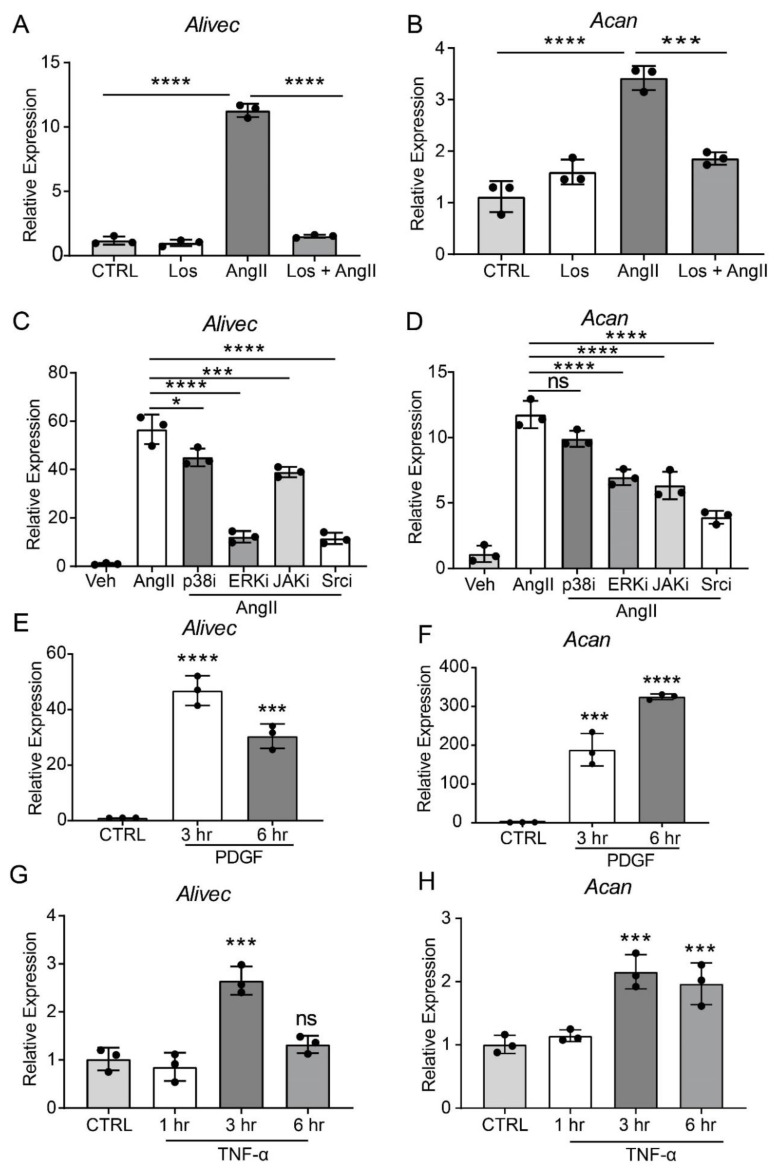
AngII-induced *Alivec* expression is regulated by AT1R and downstream kinases Src and ERK1/2. (**A**,**B**) RT-qPCR analysis of *Alivec* and *Acan* expression in RVSMCs pre-treated with the AT1R inhibitor Losartan (Los, 10 µM) for 30 min, followed by AngII treatment (100 nM, 3 h). (**C**,**D**) RVSMCs were pre-treated with vehicle DMSO (Veh) or inhibitors (i) of p38, ERK1/2, JAK and Src kinases for 30 min, followed by AngII treatment (100 nM, 3 h). (**E**–**H**) RT-qPCR analysis of *Alivec* and *Acan* expression in RVSMCs, treated with PDGF (10 ng/mL) and TNF-α (10 ng/mL). Data presented as mean ± SD. Comparisons were performed by one-way ANOVA with Tukey’s post-hoc test. (**A**–**D**) Dunnett’s multiple comparisons test (**E**–**H**), * *p* < 0.05, *** *p* < 0.001 and **** *p* < 0.0001 vs. CTRL or AngII.

AngII, through the activation of AT1R, induces multiple signaling pathways to regulate downstream gene expression and alter VSMC function [2]. To elucidate the role of AT1R signaling in regulating *Alivec* and *Acan* expression, we pre-treated RVSMCs with the AT1R antagonist, losartan (10 µM, 30 min), followed by AngII (100 nM). Losartan abrogated the AngII-mediated induction of *Alivec* (Figure 2A). *Acan* expression was also attenuated by losartan (Figure 2B). Pre-treatment of RVSMCs for 30 min with specific inhibitors of key signaling kinases, ERK1/2 (U0126, 10 µM) and Src (PP1,10 µM), significantly reduced AngII-induced *Alivec* expression. Conversely, inhibitors of the p38 MAP kinase (SB202190, 5 µM) and JAK (Inhibitor I, 10 µM), had minimal effect on *Alivec* expression (Figure 2C). Interestingly, the AngII-mediated induction of *Acan* was significantly suppressed by inhibition of the Src kinase, and to a lesser extent, by inhibitors of ERK1/2 and JAK (Figure 2D). These results demonstrate key roles of AT1R signaling and downstream kinases in the regulation of *Alivec* and *Acan* in RVSMCs.

We also determined if *Alivec* is induced by other VSMC growth factors, such as PDGF, and the inflammatory cytokine TNF-α. RVSMCs treated with PDGF (10 ng/mL) and TNF-α (10 ng/ml, 3 and 6 h) showed increased *Alivec* and *Acan* mRNA levels, although with varying kinetics (Figure 2E–H) suggesting a general effect. Together, these results show that *Alivec* and *Acan* are upregulated by not only AngII, but by other growth factors and inflammatory cytokines that might co-operate to augment VSMC dysfunction upon vascular injury.

### 3.3. Alivec Knockdown Attenuates AngII-Induced Upregulation of Genes Associated with Chondrogenesis

**Figure 3:**
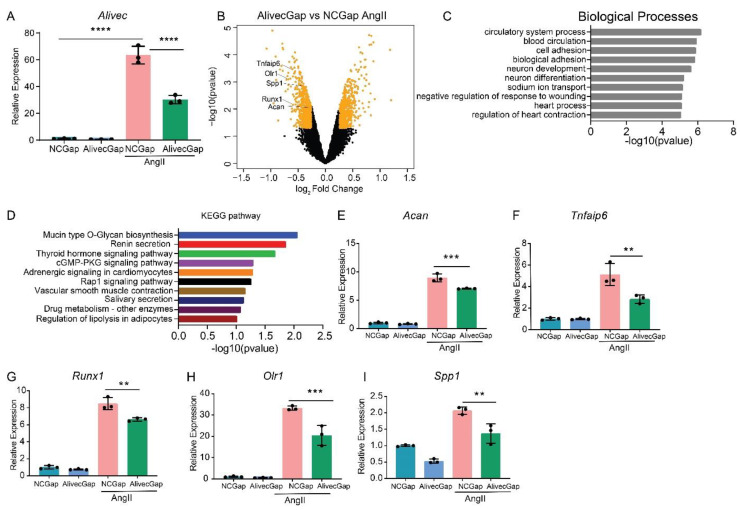
*Alivec* knockdown attenuates upregulation of AngII-induced chondrogenic genes in RVSMCs. (**A**) Knockdown efficiency of LNA GapmeR targeting *Alivec* (*Alivec*Gap) (100 nM) vs. non-targeting control GapmeR (NCGap) (100 nM), as determined by RT-qPCR. Data presented as mean ± SD, one-way ANOVA followed by Tukey’s post-hoc test and **** *p* < 0.0001 vs. indicated groups. (**B**) Volcano plot showing differentially expressed genes (orange color) in AngII-treated RVSMCs transfected with *Alivec*Gap vs. NCGap. Labeled dots indicate genes involved in chondrogenesis. (**C**) Gene ontology (GO) analysis by the TOPPGENE tool of differentially-expressed (DE) genes showing the top 10 biological processes enriched in downregulated genes after *Alivec* knockdown. (**D**) KEGG pathway analysis of differentially-expressed (DE) genes, showing the top 10 molecular pathways affected in downregulated genes after *Alivec* knockdown. (**E**–**I**) RT-qPCR validation of indicated chondrogenic genes after *Alivec* knockdown in RVSMCs treated ± AngII (100 nM, 3 h). Data presented as mean ± SD, one-way ANOVA followed by Tukey’s post-hoc test and ** *p* < 0.01 and *** *p* < 0.001 vs. indicated groups) *n* = 3 biological replicates.

AngII regulates the expression of inflammatory, fibrotic and calcification-related genes in VSMC [2]. To further characterize the role of *Alivec* in the regulation of AngII-induced gene expression in RVSMCs, a locked nucleic acid (LNA)-modified anti-sense oligonucleotide (ASO) GapmeRs-mediated knockdown of *Alivec* was performed. Three GapmeRs targeting *Alivec* were tested to assess their efficacy in RVSMCs (Supplementary Figure S2A). Results showed that GapmeR3 (denoted as *Alivec*Gap) achieved maximum reduction (~60%) in AngII-induced *Alivec* expression, as compared to the control GapmeR (NCGap) (Figure 3A and Supplementary Figure S2B). RVSMCs were transfected with *Alivec*Gap or NCGap and treated with or without AngII. RNA extracted from these cells was subjected to microarray expression profiling (Supplementary Figure S3A,B). After *Alivec* knockdown, we identified 1169 differentially expressed genes in untreated RVSMCs (676 downregulated and 493 upregulated), and 1294 differentially expressed genes in AngII-treated RVSMCs (664 downregulated and 630 upregulated), which included several chondrogenic genes (Figure 3B). Gene ontology (GO) analysis of downregulated genes showed enrichment of biological processes, such as cell adhesion and the circulatory system (Figure 3C), which are important functions of VSMC and the cardiovascular system. The Kyoto Encyclopedia of Genes and Genomes (KEGG) analysis showed enrichment of pathways involved in mucin type O-glycan biosynthesis, nitric oxide second messenger cGMP signaling and vascular smooth muscle contraction (Figure 3D) that could be associated with VSMC functions and hypertension. 

RT-qPCR validation of microarray data confirmed downregulation of *Acan* and several other chondrogenic genes, including *Tnfaip6*, *Runx1*, *Olr1* and *Spp1* (Figure 3E–I), after *Alivec* knockdown in RVSMCs. In addition, *Acan* downregulation is consistent with the known role of lncRNAs in regulating adjacent genes (Figure 3B). 

**Figure 4:**
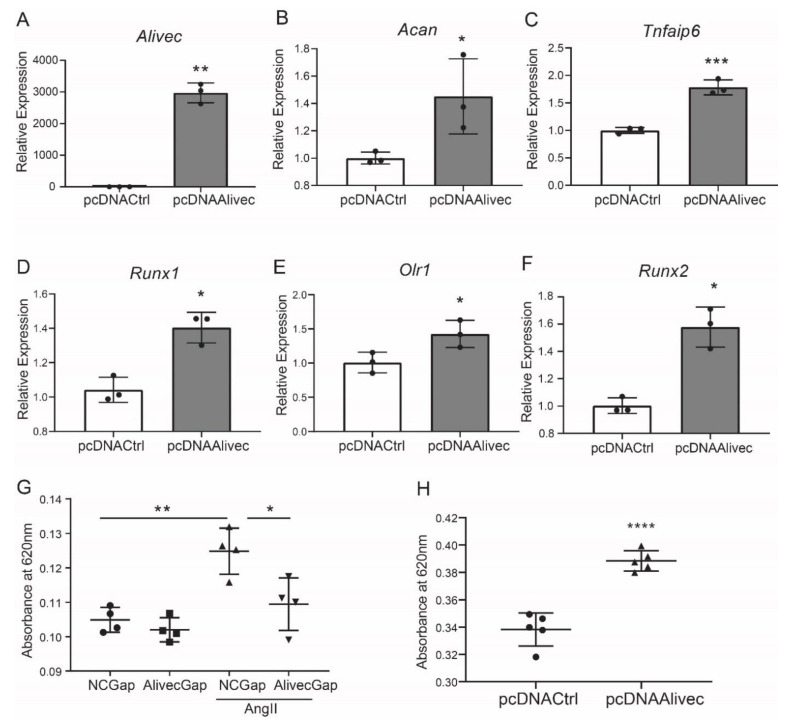
*Alivec* overexpression promotes and its knockdown inhibits the chondrogenic/osteogenic phenotype in RVSMCs. (**A**) RT-qPCR analysis showing expression of *Alivec* after transfection of RVSMC with pcDNAAlivec vs. empty vector (pcDNACtrl). (**B**–**F**) RT-qPCR analysis showing expression of target genes *Acan*, *Tnfaip6*, *Runx1*, *Olr1* and *Spp1* after overexpression of *Alivec* in RVSMCs. Data presented as mean ± SD, n = 3 biological replicates, unpaired two-tailed Student’s *t*-test and * *p* < 0.05, ** *p* < 0.01, *** *p* < 0.001 vs. pcDNACtrl. (**G**) Alcian blue staining performed on RVSMCs transfected with NCGap and *Alivec*Gap and treated ± AngII (100 nM). Data were presented as mean ± SD, *n* = 4 biological replicates, one-way ANOVA followed by Tukey’s post-hoc correction and * *p* < 0.05, ** *p* < 0.01 vs. indicated groups. (**H**). Alcian blue staining after overexpression of *Alivec* in RVSMCs. Data presented as mean ± SD, *n* = 5 biological replicates, unpaired two-tailed Student’s *t*-test and **** *p* < 0.0001 vs. pcDNACtrl.

Conversely, in gain-of-function experiments, transient overexpression of *Alivec* increased mRNA levels of *Acan*, *Runx1*, *Tnfaip6*, *Olr1* and *Runx2*, relative to the controls (Figure 4A–F). Together, these results demonstrate that lncRNA *Alivec* plays a key role in the regulation of AngII-induced chondrogenic genes in RVSMCs.

### 3.4. Alivec Mediates a Chondrogenic/Osteogenic Phenotype in RVSMCs 

Alcian blue stains glycosaminoglycan proteins, including aggrecan, that are associated with the ECM and chondrogenic differentiation [33]. Relative to control, AngII-treated RVSMCs showed increased alcian blue staining, and this was significantly decreased by *Alivec* knockdown with GapmeR (Figure 4G). Conversely, overexpression of *Alivec* increased alcian blue staining (Figure 4H). These data demonstrate that *Alivec* regulates expression of several AngII-induced chondrogenic genes, including nearby *Acan*, and promotes a chondrocyte phenotype in RVSMCs.

### 3.5. Transcription Factor Sox9 Controls Alivec Expression in RVSMCs

**Figure 5:**
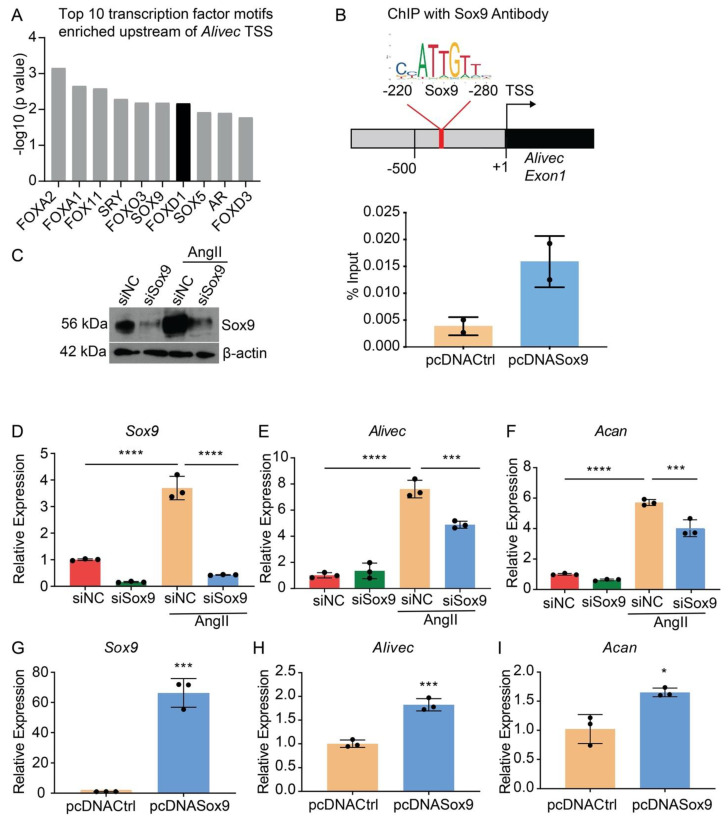
Transcription factor Sox9 controls *Alivec* expression in RVSMCs (**A**). Top 10 transcription factor (TF) binding motifs, enriched in the genomic region upstream of *Alivec* transcription start site (TSS). (**B**) ChIP assays with Sox9. Upper panel depicts schematic of the predicted Sox9-binding site upstream of *Alivec* TSS (arrow). Lower panel show ChIP-qPCR data with Sox9 antibody in RVSMCs transfected with pcDNACtrl and pcDNASox9 plasmids. ChIP-DNA was analyzed by qPCR with *Alivec* promoter primers overlapping Sox9-binding sites (*n* = 2). (**C**) Sox9 knockdown with siRNAs. Sox9 protein levels determined by Western blotting in RVSMCs transfected with siRNA targeting *Sox9* (siSox9) or negative control (siNC) oligonucleotides, and treated ± AngII (upper panel). β-actin protein levels (lower panel) were used as internal control. (**D**–**F**) RT-qPCR analysis of indicated genes in siSox9- and siNC-transfected RVSMCs at the basal level and after stimulation with AngII. Data presented as mean ± SD, *n* = 3 biological replicates, one-way ANOVA followed by Tukey’s post-hoc test and *** *p* < 0.001, **** *p* < 0.0001 vs. indicated groups. (**G**–**I**). RT-qPCR analysis of indicated genes in RVSMCs, transfected with Sox9 expression plasmid (pcDNASox9) and pcDNACtrl control plasmid. Data represented as mean ± SD, *n* = 3 biological replicates, unpaired Student’s *t*-test and * *p* < 0.05, *** *p* < 0.001 vs. control plasmid.

Transcription factor (TF) motif analyses of 500 bases upstream of the *Alivec* transcription start site (TSS) showed enrichment of ten TFs, including Sox9 (Figure 5A). Sox9 regulates chondrogenesis and osteogenesis in mesenchymal stem cells [34]. We examined Sox9 interaction with the *Alivec* promoter in RVSMCs transfected with a Sox9 expression plasmid (pcDNASox9) and a control vector (pcDNACtrl), using chromatin immunoprecipitation (ChIP) assays with the Sox9 antibody. ChIP-qPCR showed enrichment of Sox9 in the predicted Sox9-binding region, upstream of the *Alivec* TSS, as compared with the control pcDNACtrl plasmid-transfected cells (Figure 5B). Transfection of RVSMCs with the siRNAs targeting *Sox9* (siSox9), reduced the Sox9 protein and transcript levels in control- and AngII-treated cells (Figure 5C,D). Sox9 knockdown also decreased the AngII-induced expression of *Alivec* and *Acan* (Figure 5E,F). Conversely, the overexpression of Sox9 using the pcDNASox9 plasmid in RVSMCs increased *Alivec* and *Acan* vs. the control vector-transfected cells (Figure 5G–I). These results demonstrate that Sox9 can regulate *Alivec* and *Acan* expression in response to AngII in RVSMCs.

### 3.6. Alivec RNA Interacts with hnRNPA2B1 as well as with Tropomyosin alpha-3 Chain, a Protein with Putative Association with the Contractile Phenotype of RVSMCs

**Figure 6:**
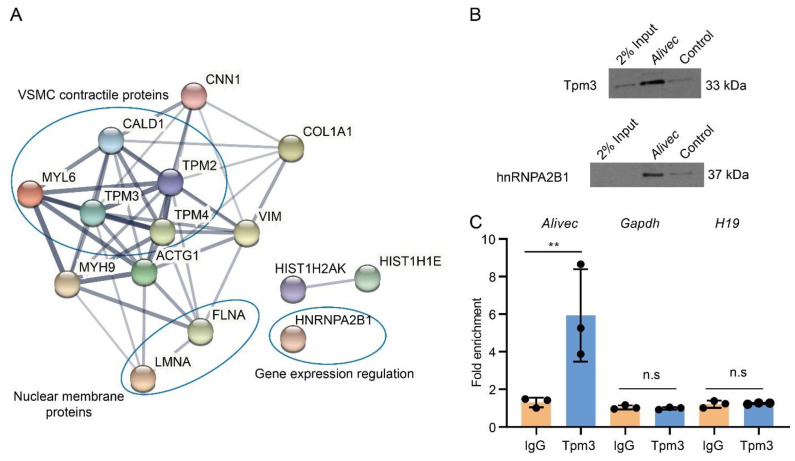
*Alivec* RNA interacts with hnRNPA2B1 and tropomyosin-3-alpha, a protein potentially associated with the contractile phenotype of VSMCs. (**A**) Network of protein complexes generated (using STRING database) from *Alivec*-specific interacting proteins identified by RNA-pulldown coupled to mass spectrometry. (**B**) Western blot analysis with Tpm3 antibody (upper panel) or hnRNPA2B1 antibody (lower panel) following RNA-pulldown assays with RVSMCs extracts using biotinylated *Alivec* RNA and poly A RNA as negative control (Control). (**C**) RNA immunoprecipitation assays with UV-cross-linked RVSMC cell extracts using anti-Tpm3 antibody and IgG as negative control. RNA from Tpm3 and IgG immunoprecipitates were analyzed by RT-qPCR, using indicated primers. Results were shown as fold enrichment over IgG. Data presented as mean ± SD, *n* = 3 biological replicates and ** *p* < 0.01 vs. IgG, using unpaired Student’s *t*-test. N.s. indicates not significant.

LncRNAs can regulate transcription, gene expression and cellular phenotype through interactions with proteins [35,36]. We performed RNA-pulldown assays with *Alivec*, followed by mass spectrometry, and found a number of proteins associated with *Alivec*, relative to negative control. STRING analysis demonstrated that the *Alivec* interacting proteins were associated with VSMC contractile functions, nuclear membrane organization and regulation of gene expression (Figure 6A). One of these proteins, a tropomyosin alpha-3 chain (Tpm3) [37] was noteworthy, due to the known roles of alpha-tropomyosin isoforms in VSMC contractile functions and gene regulation [38,39]. RNA–protein interaction prediction (RPISeq) software showed that the *Alivec*-Tpm3 RNA–protein interaction had a positive interaction probability of 0.75 (>0.5 considered positive). We then performed RNA-pulldown, followed by Western blot analysis, in order to validate the Tpm3 association with *Alivec* (Figure 6B), which confirmed our mass spectrometry results. Specific interaction of *Alivec* with Tpm3 was also supported by RNA-immunoprecipitation (RNA-IP), using an antibody against Tpm3. No interaction was seen with *Gapdh* mRNA and *H19* lncRNA (negative controls, Figure 6C). In addition, mass spectrometry showed that *Alivec* interacts with the RNA-binding protein, heterogeneous nuclear ribonucleoproteinA2B1 (hnRNPA2B1), which was validated by RNA-pulldown, followed by Western blotting (Figure 6B, lower panel). These results indicate that lncRNA *Alivec* mediates AngII-induced VSMC dysfunction via interaction with both cytoplasmic and nuclear proteins.

### 3.7. AngII Treatment Increases Aortic Expression of Alivec in Rats

**Figure 7:**
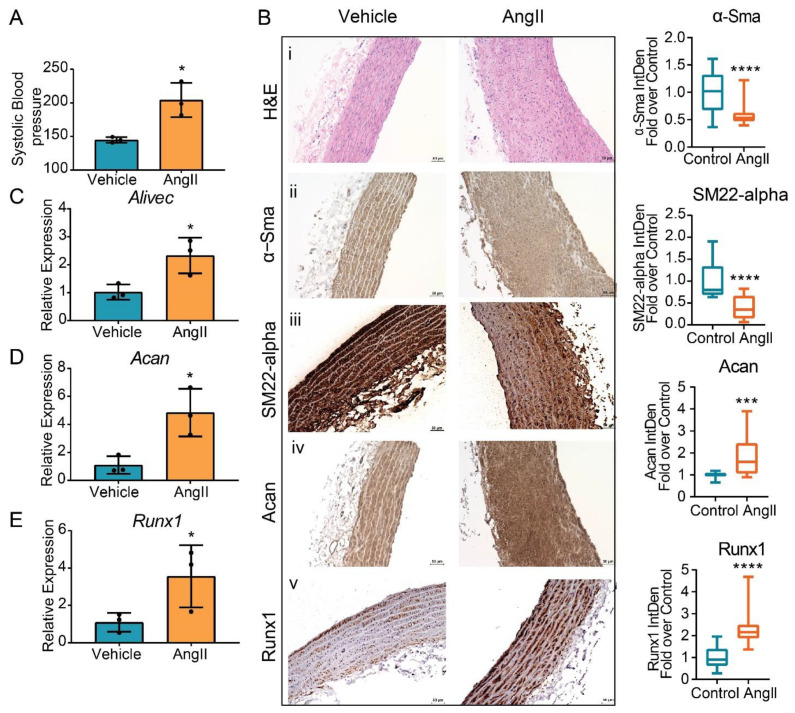
Regulation of *Alivec* and *Acan* in the aortas from a rat model of AngII-induced hypertension. (**A**) Systolic blood pressure (SBP) measured in male Sprague–Dawley rats infused with AngII or vehicle for 4 weeks. (**B**) Representative images of hematoxylin and eosin (H&E) staining (i) and IHC staining for α-Sma (ii), SM22-alpha (iii), Acan (iv) and Runx1 (v) proteins on aortic tissue sections from vehicle or AngII-infused rats, scale bar: 50 µM. Box plots on the right show the quantification of aortic staining of indicated proteins shown in panels (ii) to (v). Box plots on the right show integrated density (IntDen) expressed as fold-over control. Staining was quantified using ImageJ software in 20 different areas for each group (3 aortas in control and 3 in AngII group. Data represented as mean and minimum/maximum, unpaired Student’s *t*-test and *** *p* < 0.001 and **** *p* < 0.0001). (**C**–**E**) RT-qPCR analysis showing gene expression of *Alivec*, *Acan* and *Runx1* in aortas from AngII-infused rats in comparison to vehicle-treated rats. Data presented as mean ± SD, *n* = 3 biologic replicates and unpaired Student’s *t*-test. * *p* < 0.05 vs. vehicle.

Next, we examined whether AngII upregulates the expression of *Alivec* and *Acan* in vivo in rats. Male Sprague–Dawley rats (12-weeks-old), infused with AngII (200 ng/kg/min, four weeks), showed the expected increase in systolic blood pressure (SBP) compared to control vehicle (PBS) infused rats (Figure 7A). Aortic thickening was noted in AngII-infused animals relative to the controls (Figure 7B). Immunohistochemical staining showed marked increases in aggrecan and Runx1 proteins and decreases in the smooth muscle contractile proteins α-SMA and SM22 alpha (Figure 7B). Furthermore, mRNA levels of *Alivec*, *Acan* and *Runx1* were significantly increased in vessels from the AngII group (Figure 7C–E). 

### 3.8. The Human ALIVEC Locus Contains ACAN Regulatory Elements and a Blood Pressure Quantitative Trait Locus (QTL)

**Figure 8:**
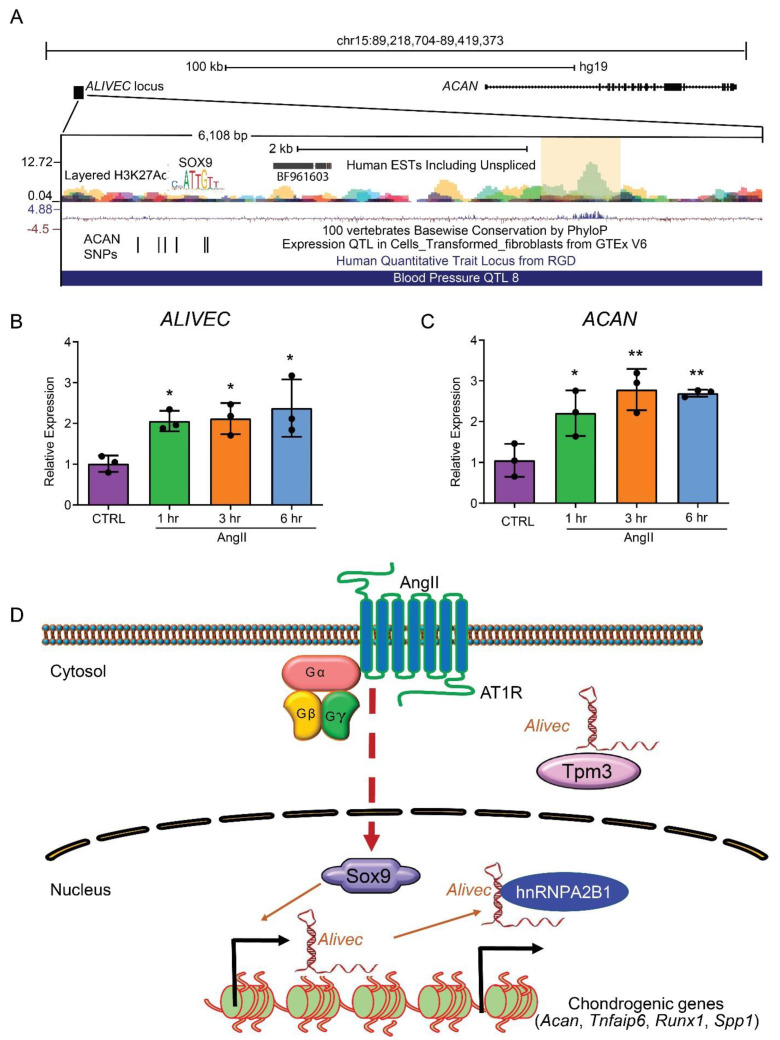
The human *ALIVEC* locus contains *ACAN* regulatory elements and a blood pressure quantitative trait locus (QTL). (**A**) UCSC human genome browser tracks showing *ACAN* to the right, *ALIVEC* locus to the left and is enlarged showing BF961603 EST (potential *ALIVEC*), *ACAN* regulating enhancer (light yellow shaded region), expression QTLs (eQTLs) that regulate *ACAN* expression and a blood pressure-associated QTL 8, stretching through *ALIVEC* locus. (**B**,**C**) HVSMCs were treated with ± AngII (100 nM) for the indicated time periods and RT-qPCR analysis of *ALIVEC* and *ACAN* expression was performed. Data presented as mean ± SD, *n* = 3 biological replicates and one-way ANOVA with Dunnett’s multiple comparisons test. (* *p* < 0.05, ** *p* < 0.01 vs. CTRL. CTRL indicates control). (**D**) Schematic model depicting the role of *Alivec* in AngII-induced VSMC chondrogenic transition. In RVSMCs, AngII induces lncRNA *Alivec* via activation of AngII type 1 receptor (AT1R) and downstream transcription factor Sox9, a master regulator of chondrogenesis. In turn, *Alivec* localized in the nucleus modulates Sox9-induced expression of chondrogenic genes, such as nearby *Acan* potentially through enhancer activity, and distantly localized *Tnfaip6*, *Runx1* and *Spp1* via trans-acting mechanisms to promote chondrogenesis. Interaction with nuclear proteins, such as hnRNPA2B1 may play a role in *Alivec* mediated gene regulation. Whereas, interactions in the cytoplasm of *Alivec* with Tpm3 proteins may disrupt contractile functions of VSMC. Thus, *Alivec* may play an important role in AngII-induced RVSMC phenotypic, switching from contractile to pathologic phenotypes associated with hypertension and CVDs.

Using a syntenic approach through the UCSC genome browser LiftOver tool, we observed a putative orthologous *Alivec* locus in the human genome on chromosome 15, adjacent to *ACAN* (Figure 8A) [40]. This locus consists of an expressed sequence tag (EST) BF961603, that may represent the human ortholog, *ALIVEC* (Figure 8A). This EST is 514 nucleotides in size and does not have coding potential with a probability of 0.00511, as assessed by CPC2 analysis. Similar to the rat *Alivec* locus, the human locus has H3K27ac peaks (shaded region) that are predicted to be an *ACAN*-regulating enhancer element based on GeneHancer (GeneCards) regulatory elements in the UCSC genome browser. This enhancer locus has a higher PhyloP conservation score, suggesting a similarity between the rat and human genome [41]. The upstream genomic region of *ALIVEC*-EST possesses a SOX9-binding site similar to the *Alivec* upstream region in rats. Interestingly, the human *ALIVEC* locus is part of a QTL associated with blood pressure, identified from genetic analysis of inherited hypertension in rats and by further genome lift-over to human genomes [42]. It harbors six expression QTLs (eQTLs) that regulate ACAN expression (Figure 8A) [43]. Moreover, we found that AngII-treated human VSMCs (HVSMCs) showed increased expression of *ALIVEC* (Figure 8B) and *ACAN* (Figure 8C), relative to controls. These results suggest that the putative human *ALIVEC* gene locus and transcript have similar features and functions as the rat *Alivec*.

## 4. Discussion

LncRNAs are important regulators of VSMC function and play key roles in the development of CVDs [16,23]. Hypertensive animals show loss of a VSMC contractile phenotype and gain of a chondrogenic phenotype [44]. This study is the first to identify a lncRNA (*Alivec)* that regulates the expression of the AngII-induced genes known to be involved in the trans-differentiation of VSMCs to chondrocyte-like cells. We found that *Alivec* and its neighboring protein-coding gene *Acan* (usually associated with chondrocytes) are induced by AngII as well as by other stimuli known to promote VSMC de-differentiation. Furthermore, in RVSMCs, AngII induces *Alivec* through AT1R via Src and ERK1/2 and TF Sox9. Gain of function and knockdown approaches clearly demonstrated that *Alivec* can modulate AngII-induced chondrogenic genes. Altogether, our study suggests a role for *Alivec* in promoting the chondrogenic differentiation of VSMCs. 

Aggrecan (Acan), also known as chondroitin sulfate proteoglycan 1, is encoded by the *Acan* gene (*ACAN* in humans) [45,46]. Acan protein is an integral part of the extracellular matrix in cartilage. It is increased with vascular injury and is associated with hypertension [47,48]. TNF-α-induced protein-6 (TSG-6), a protein product of *TNFAIP6*, and ACAN protein interact to promote matrix stability [49]. The oxidized low-density lipoprotein receptor 1 (OLR1), also known as LOX1, has a pro-inflammatory role. *Olr1* is induced by AngII and is associated with phenotypic changes in VSMCs and hypertension [50,51]. Osteopontin, a multifunctional glycophosphoprotein protein, is encoded by the *SPP1* gene (secreted phosphoprotein 1). Osteopontin is produced by VSMCs, endothelial cells and macrophages, and plays an important role in physiological and pathophysiological processes, including the regulation of biomineralization and VSMC calcification [52,53,54]. The role of lncRNAs in regulating these genes in VSMCs is not known. We found that the knockdown of *Alivec* led to a reduction of AngII-induced *Alivec* and *Acan*, as well as the chondrocyte-associated genes *Tnfaip6*, *Runx1*, *Olr1* and *Spp1*. Furthermore, as indicated by alcian blue staining, AngII treatment promoted a chondrocyte-like phenotype in VSMCs, which was attenuated with *Alivec* knockdown and increased by *Alivec* overexpression, implicating a functional role for *Alivec* in chondrogenic transformation of VSMCs. 

Moreover, aortas from AngII-treated hypertensive rats displayed increased expression of *Alivec* and *Acan* along with reduced expression of contractile genes. These findings suggest that *Alivec* upregulation occurs under conditions of hypertension and aortic wall remodeling. Relevant to this, VSMCs, chondrocytes and osteoblasts are all derived from mesenchymal stem cells [55]. Moreover, arterial injury in SM22 knockout mice resulted in VSMC transformation to chondrocyte-like cells [31]. In light of these new and published findings, rodent models of atherosclerosis and chronic hypertension may reveal unique interactions between plaque formation, arterial remodeling, vascular calcification and *Alivec*. 

Our examination of lncRNA-binding proteins provided key insights into the mechanisms of action and regulation of the target genes by *Alivec* in VSMC. We previously showed that interaction of AngII-induced lncRNA *Giver* with the RNA-binding protein NONO played a role in regulating genes associated with inflammation and oxidative stress in VSMCs [23]. Herein, mass spectrometry analyses of RNA-pulldown experiments identified Tpm3 and hnRNPA2B1 as major *Alivec* interacting proteins. STRING analysis of these and other *Alivec* interacting protein-binding partners provided clues regarding potential mechanisms, through which *Alivec* regulates target gene expression and enhances the chondrocyte phenotype of VSMCs. Tropomyosins are cytoskeletal proteins that regulate smooth muscle cell contraction through interaction with actin. Levels of tropomyosin 1 (Tpm1) protein were downregulated in response to high glucose in VSMCs, and this augmented VSMC transition to a synthetic phenotype [56,57]. It is possible that AngII, by increasing cytosolic *Alivec*, could sequester Tpm3 and inhibit its functions, leading to reduction in the contractile features of VSMCs, while increasing their synthetic and chondrogenic features. Concurrently, nuclear *Alivec*, through interactions with hnRNPA2B1, might regulate other target genes in trans, including chondrogenic genes. *Alivec* overlaps an enhancer, suggesting it could potentially be an enhancer-RNA (eRNA) and may also regulate the neighboring gene *Acan* through enhancer activity. But further in-depth studies are needed to determine the enhancer effects of the *Alivec* locus and *Alivec*’s function as eRNA in VSMCs. *Spp1* is a target gene of *Alivec* that we identified and hnRNPA2B1 is involved in the regulation of *Spp1* expression in macrophages [58]. Similar to *Alivec*, *lincRNA-Cox2* is localized in the nuclear and cytoplasmic compartments of macrophages [59]. Nuclear *lincRNA-Cox2* interacts with hnRNPA2B1 and regulates the expression of immune genes in response to activation of toll-like receptor signaling [59]. Together these data suggest that *Alivec* acts via nuclear hnRNPA2B1 and cytoplasmic Tpm3 to alter gene expression and phenotype. However, additional mechanistic studies, including determining the direct functions of Tpm3 and hnRNPA2B1 in VSMCs, are needed to confirm this. 

Of translational relevance, we identified a potential human ortholog of *ALIVEC* in AngII-treated HVSMCs. Interestingly, this *ALIVEC* locus is part of a QTL associated with blood pressure. Identification of this QTL was based on the genetic analysis of inherited hypertension in rats and by further genome lift-over to humans [42]. However, the function of these variants and their association with human hypertension, has not been determined. In addition, ATAC-seq data from the transforming growth factor (TGF)-β-treated human coronary artery SMCs, identified an inducible open chromatin region in the enhancer region of the *ALIVEC* locus (Supplementary Figure S4) [60]. These data suggest, similar to the rat locus, the presence of an active enhancer element in the *ALIVEC* locus of the human genome that is responsive to TGF-β and PDGF. Furthermore, the presence of open chromatin in this region, along with the H3K27ac peak predicted as an *ACAN* regulating enhancer, supports connections between *ALIVEC*, VSMC chondrogenic-like phenotype and blood pressure. Furthermore, an EST in this region was also induced by AngII in HVSMCs. However, additional studies are needed to fully characterize the putative orthologous human transcript and determine its potential connections to human hypertension. 

Limitations of the study include the paucity of details on how *Alivec*-interacting proteins modulate VSMC function, as well as the inadequate characterization of the putative human transcript and the functional relationship to AngII-induced hypertension. Additional mechanistic studies are required to elucidate the nuclear vs. cytoplasmic actions of lncRNA *Alivec*. Examination of remodeled and calcified arteries of hypertensive rodents and humans may help in identifying *Alivec* as a potential biomarker for CVD development and progression. Nonetheless, the data presented demonstrate that a novel AngII-induced lncRNA *Alivec* regulates genes associated with the VSMC phenotypic transition to chondrocyte-like cells and is likely associated with blood pressure regulation. These results provide new insights into the role of lncRNAs in chondrogenesis and key pathologic vascular actions of AngII that could lead to new therapeutic targets for AngII-regulated CVDs. 

## 5. Conclusions

Together these results demonstrate that a novel AngII induced lncRNA *Alivec* regulates genes associated with chondrogenic transformation of VSMCs implicated in vascular dysfunction, which could lead to the identification of non-coding RNA based biomarkers and therapeutic targets for CVDs.

## Data Availability

Microarray expression datasets are deposited in GEO with accession (GSE183857).

## Supplementary Materials

The following are available online at https://www.mdpi.com/article/10.3390/cells10102696/s1, Figure S1: *Alivec* characterization and full-length cloning. Figure S2: Design and efficacy of LNA-GapmeRs targeting *Alivec*. Figure S3: Microarray profiling in RVSMCs transfected with NCGap or AlivecGap. Figure S4: Chromatin accessibility at the putative human *ALIVEC* locus in human coronary artery smooth muscle cells (HCASMCs). Table S1: Primer sequences used in the study. Table S2: Complete *Alivec* sequence. Table S3: GapmeRs and siRNA sequences. Table S4: Antibodies used in the study and their source.
