# Supplementary material for: Angiotensin II-Induced Long Non-Coding RNA Alivec Regulates Chondrogenesis in Vascular Smooth Muscle Cells
Source: Cells. 2021 Oct 9;10(10):2696. doi: 10.3390/cells10102696 (PMC8535098; doi:10.3390/cells10102696)
Supplement: Supplementary file 1 [file cells-10-02696-s001.zip › cells-1403573-supplementary.pdf]

Supplementary Materials (Figures and Tables) for Vishnu Amaram Samara, et al.

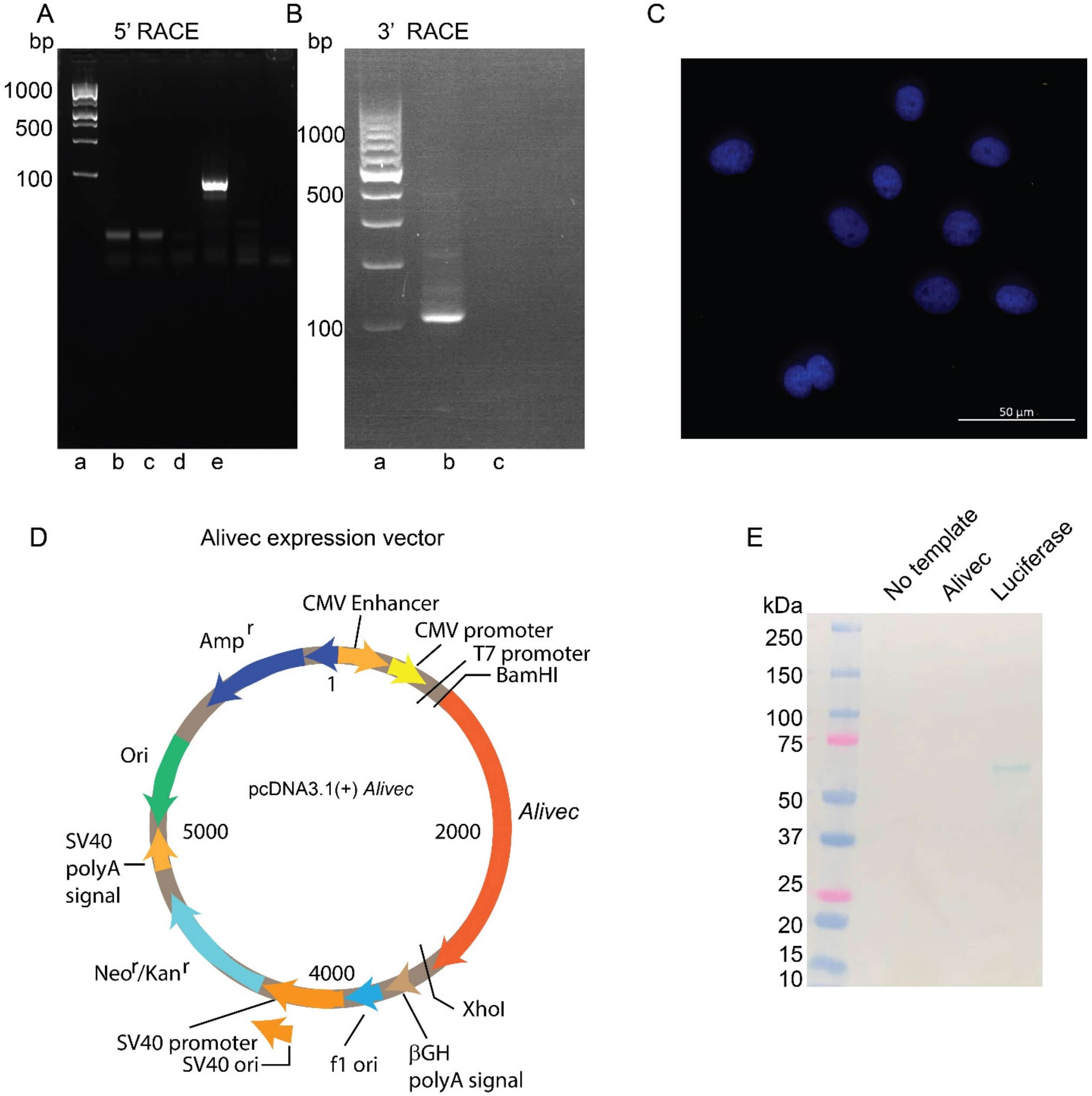

Supplementary Figure S1. ***Alivec*** **characterization and full-length cloning**

A) Representative agarose gel image of 5’ RACE of *Alivec* (lane a -100bp DNA ladder, b - no template PCR, c - 5' outer *Alivec* RACE-PCR, d - empty lane, e - 5’ inner *Alivec* RACE-PCR that was chosen for Sanger sequencing. (the faint bands at lower mol wt. of ~20bp in lanes b and c are artifacts that could be from primer dimers or reaction mixture) B) Representative agarose gel image of 3’ end RACE products of *Alivec* (a – 100 bp DNA ladder, b – 3’*Alivec* RACE-PCR chosen for

Sanger sequencing, c – no template PCR). C) Control image of RNA-FISH with no probes showing absence of spots in VSMC; nuclei are stained with DAPI (blue), Scale bars represent 50μm. D) Plasmid map of *Alivec* sequence cloned into pcDNA3.1+ overexpression vector to generate pcDNA*Alivec* plasmid. E) *In vitro* transcription/translation assay with pcDNA*Alivec* plasmid performed using T7 TNT Quick coupled transcription/translation system (Promega). T7 luciferase plasmid was used as positive control and no template pcDNA plasmid as negative control.

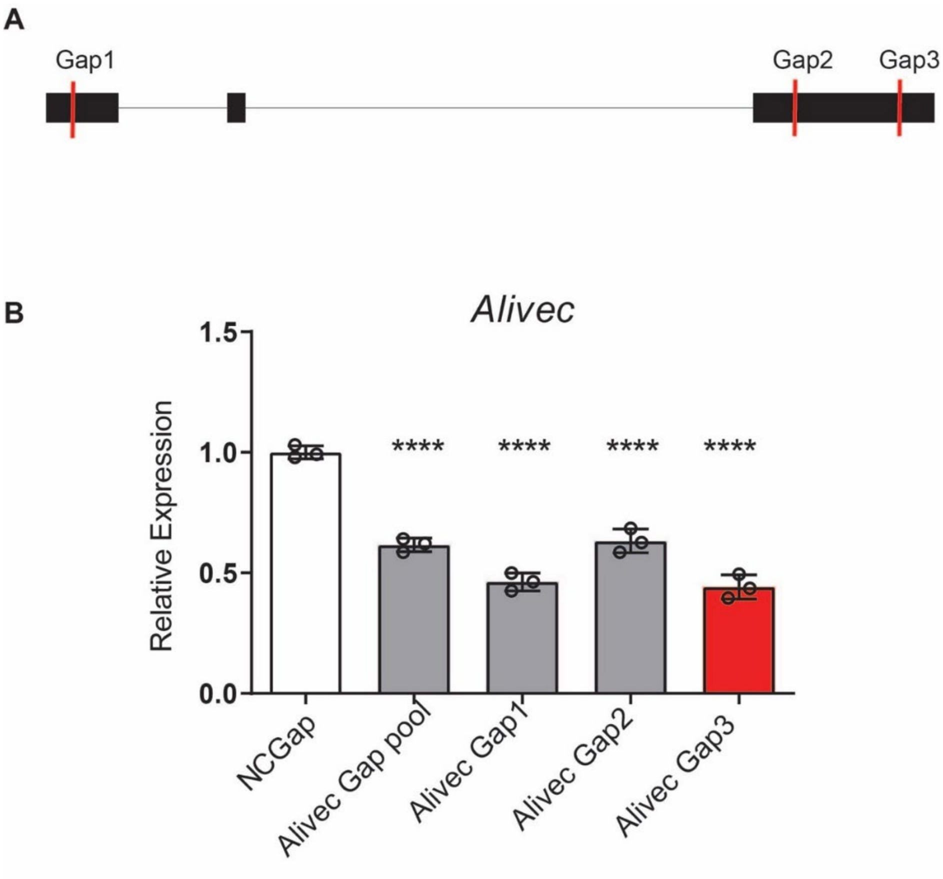

Supplementary Figure S2. **Design and efficacy of LNA-GapmeRs targeting *Alivec***

A) Three GapmeRs (Gap1, Gap2, Gap3) were designed using the Qiagen-Exiqon GapmeR designer tool to target three indicated regions of the *Alivec* transcript sequence. B) RT-qPCR analysis of *Alivec* in RVSMCs 48 hours post transfection with GapmeRs targeting *Alivec* or non-targeting GapmeR (NCGap) at 100nM. Individual GapmeRs and a pool of these GapmeRs were tested for knockdown efficiency of *Alivec* in the presence of Ang II (100nM, 3 hour). AlivecGap3 was most effective and was used for subsequent knockdown experiments (AlivecGap). Data presented as mean ± SD, n=3 biological replicates, one-way ANOVA with Dunnett's multiple comparisons test, ****$p<0.0001$ versus NCGap.

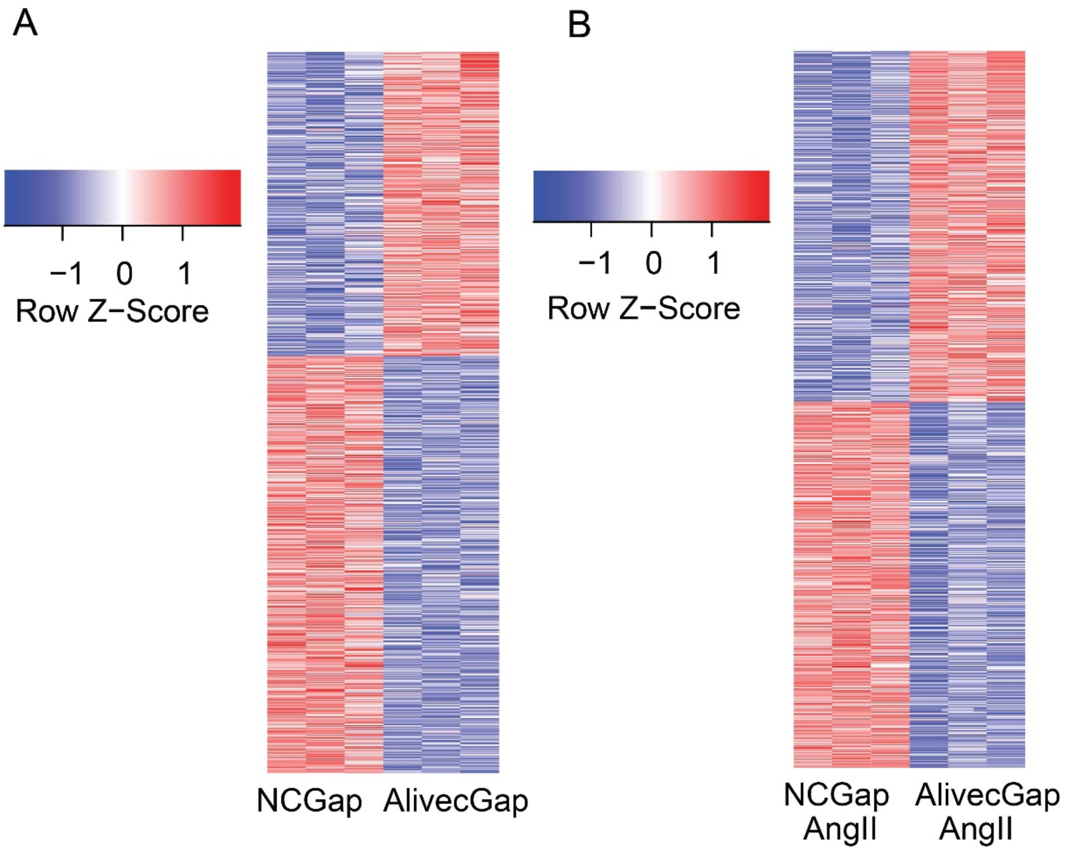

Supplementary Figure S3. Microarray profiling in RVSMCs treated with NCGap and AlivecGap

A, B) Heat map of differentially expressed genes from RVSMCs transfected with AlivecGap or NCGap and treated without (A) or with (B) Ang II (100 nM). n=3 per group. Cut off value: fold change >=1.20, p value <0.05

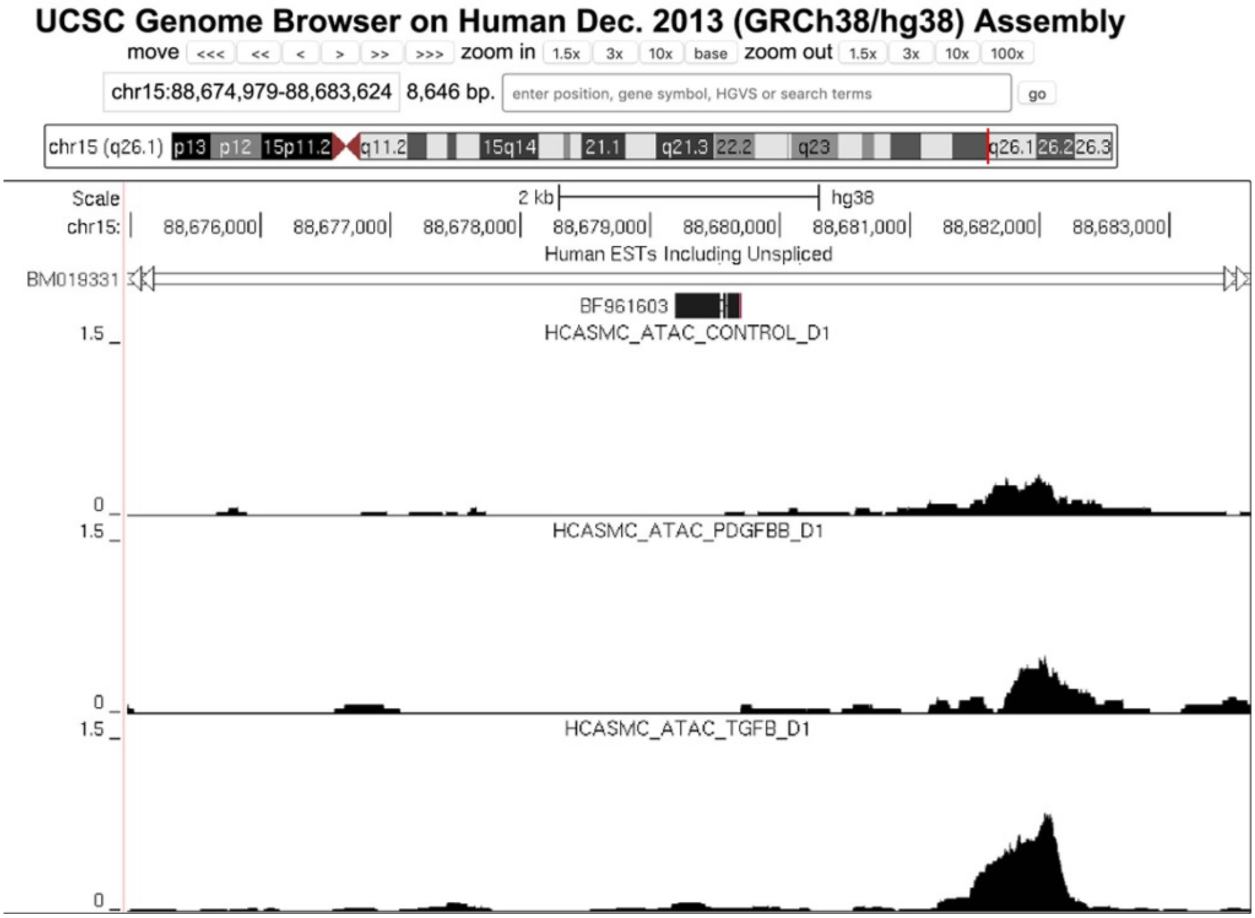

Supplementary Figure S4. Chromatin accessibility at putative human *ALIVEC* locus in Human Coronary Artery Smooth Muscle Cells (HCASMCs)

Normalized ATAC-seq tracks generated from HCASMCs under control, and PDGF-BB or TGFB treated conditions. Tracks were adapted through UCSC genome browser from https://pubmed.ncbi.nlm.nih.gov/27386823/. ATACseq represents Assay for Transposase-Accessible Chromatin using sequencing, PDGFBB and TGFB indicates platelet derived growth factor and transforming growth factor-beta.

Supplementary Table S1. Primers sequences used in the study

**Rat gene qPCR primers**	Forward	Reverse
Alivec	CTGCCCTTCTTTTCACCATGC	GCGGTACACCTGAAGCTAGT
Acan	ACTCAGGACATTAGCTGCATG	CACGTGTTCCCATTCACTTTG
Runx1	CGGGCAATGACGAAAACTAC	GGGTTTGTAAAGACGGTGATG
Spp1	TGGCTTACGGACTGAGGTCA	GACCTCAGAAGATGAACTCT
Tnfaip6	GTAGGAAGATACTGCGGTGATGAA	GACGGACGCATCACTCAGAA
Sox9	AGGAAGCTGGCAGACCAGTA	ACGAAGGGTCTCTTCTCGCT
Olr1	ACGAGAAATCCAAAGAGCAGG	TCCCATTCAGCTTCCAGTTG
Ppia	TATCTGCACTGCCAAGACTGAGG	CTTCTTGCTGGTCTTGCCATCC
Neat1	GCAAGACCATGTGCCCTAGT	TCTGGAATTGGCCGGAAGTC
H19	TTGTCTGGCTTCATCCTGTG	TGGACTTGGTGCACTGTATG
**Rat ChIP qPCR primers**		
Alivec promoter	TTGTACCTTGTCCCCAGTTG	ATGCCCAACCCCATAGTTAG
**Human gene qPCR primers**		
ALIVEC	TGCCAAGCACCACTCTCAAT	AAGGTCAATGCCAGACAGGG
ACAN	TGCGGGTCAACAGTGCCTATC	CACGATGCCTTTCACCACGAC
GAPDH	CTTTTGCGTCGCCAGCCGAG	CCAGGCGCCCAATACGACCA

Supplementary Table S2. Complete sequence of *Alivec*

ATAAAAGCCTACGGTCCTTTTGTAGACATAGTTCATTGCCAGACTGGCCTTGAATTC
GCAATCATGAGAAGACACAGGTACCTTCAGTCCAACGACTGTTGCATTAATTAGCCA
GGGTGAAACTTGGGGATGTCAAGGATAGAGAGTTAAAGTGATTTCATGAAGAAAAG
GTATTGTAGTGACTATCCCTGTTGTCAACTTGACTATCCAGAATTGGAAGGTTCACCT
GTGATCGAGGTCTTGGGGGCTGGGAGATACAAGTTTCTGACCTTGATCTTGTCATGA
AGATCTTCTATATCTGGAGTGAACTAGAGGCACAGTGGCTATGAATCCCAGGCAAG
AAGATCTCTGAGTTCAAGGTCATCTGGGACAAAGCAAGTCCCAGATCCAGAAGTCT
GGATTACACATCTTTAATCTGGGCTACACCTTCTGCTGGAGACCTACATAAGGACAT
TGGAAGAAGGGAGATGCTCTCTGCCTGCTTGCTTGCCTTGTAGGACTGAGCCACTGC
TAGATCTTTGGACTTCCAATCATAGCTGCTGCTGACCATTGTTGGGGAGTTGGACTA
CAGACTGTAAGCCGTCAACAATTTCCCTTACATATAAAGACTACTCATAAGTTCTGT
GACTCTAGAGAACCCTGACTAATATAGTGTTGCCTTCTGTTCTCGGCATCTGTCTTCA
GATTCCTATTGCTGTGATAAAACACCATGGCCAAAAGCAACTTGGAAGAAAAGGGT
TTGTGTCCCATCATCCAGGGACTTCGGCCAACAGCCAGCAGTGCAGACAAGGGCGG
CATGGACCTGCAGCCTGTGGCTGGCCCCCGAGGGCGAGCAGCCAGATGCCGGCAGC
CGCAGGAGAGAAGGGCTGCTCTTCTCTTCACTCAAAGGTGGATTGAGGAAGGTTGG
CTCTGAGATTTTTTTTCAAGAAGCTCGTCGGGAGTGGGAGAATATGAACATCTGTG
CCCTGGGTTCAGTTCTGGACTCGGCTCAGGCCACAGTCTGGAGCTGGCGTCAGCTGG
AGACAAGCCTGCATCCTGCTGGCGGGTGGGTGGGGCTGCTTCTGGCCTGCTGGTTGA
GGAACGCAGACACTAGCCTCCACCCTCTGGAGCCCAGGAGAGCGGCGGGCATGGGA
GGGCACACAGAGCTCTCAGCGCTCAGGCACCAGGGTGCCTGCATGTACTGTGTTTAC
AGAAGGGCTGGGGGCAGGTCCTGGAGTCTCGCAGGTACACTCCACAGGGAAAGGCA
CCCCAGGTCTTTCCCTCCCCCCTCTGCCTTTGGTGACAGGCATTTGTGGCCAATACTG
ATGTTCCTCTCTACACTTTATTCCCAGTCCCAGTTGTTCATCAAGGCTGCAAGACAAT
CCAGAAATTACCTAGTCAACTGGTTTTCCCCTGACCTTTGCTTTTTTTTAACTTAGCC
TATTTTTTTAACTTAAATTTTAGTGTGAAAAGGTAGTATATTTTGCTGGGGATATAGC
TCAGTTGGCAGAGGGCTTGCCTAGCATGTACCACTAGGCCCTGGATTTGAGCCCCAA
CACCACACAAACTAGGCATGTTGCTGTAAGTCAATAAACCCAATCAATACTCTGGAA
GTTCAAGATCTTCCTCAACTATCTATCCAGTTCAAAACCAGTTTAAAAGAAAACAGT

GTATTTACATGGTTCACATTTTTAAACGCTTAAATTGGTATCAATCTATATTTATATC
TATCTATATATCTATATATCATCTATATCCATCTTTATCTCCATCTCTCTCTCCTCTCT
GTCATCTATCTATATATCTCACACTTATCTGGATTTCATCTGCCCTTCTTTTCACCATG
CCTTCCTACCTCATAGCCCACAGTAAGATATGTACTAGCTTCAGGTGTACCGCTTCTT
GGCTGGGTTTGTTTAGTAGCACACACCTTTAATCTCAGTACTGGGAGGCAGAAGAAG
ACAGATCTTATGAATGTGAGGCCAGCCTGGTCTACATAGAAAGTTCTAGGCCAGCCA
AGACTACATAATGAGATTCTGTCTCTAACAAACAAATCCTTATACAGTTTTGAAATG
AAAATTGAGAATTAAAAATTTCATATCAAAAAGTAGCTTTTACTTCATTACTTTATTT
TATTATGTAATAAATTGTGTACAATATATTTTTCTATATTTTTGTTTTGTGGATAAAC
ATTATTAGCACTAAGATGCAATCCACAGACCACAGGAAGCTCAAGAAGAAGGATGA
CCAAAATGCGGATGCTCTCACTCCTTCTTAAAAGGGGCAAAAAAAAAA

Supplementary Table S3. Sequences of GapmeRs and siRNAs used in the study

GapmeR or siRNA	Sequence	Vendor or source
Alivec Gap1	GCAACAGTCGTTGGAC	QIAGEN
Alivec Gap2	TTGAACTTCCAGAGTA	QIAGEN
Alivec Gap3 (*Alivec*Gap)	GATTGCATCTTAGTGC	QIAGEN
Negative control Gap (NCGap)	AACACGTCTATACGC	QIAGEN
Sox9 siRNA pool	GCGUCAACGGCUCCAGCAA; GCUCGGAACUGUCUGGAAA; GCCAGGUGCUGAAGGGCUA; GUAAGUGAAGGUAACGAUU	Horizon

Supplementary Table S4. Antibodies used in the study and their source

Target protein	Vendor or source	Antibody & Cat No.
Tnfaip6	Proteintech	Anti-TSG6, 13321-1-AP
Runx1	Proteintech	Anti-RUNX1,19555-1-AP
Tpm3	Genetex	Anti-Tpm3, GTX113568
hnRNPA2B1	Origene	Anti-hnRNPA2B1, TA3140
Alpha-SMA	Abcam	Anti-SMA, ab5694
Tagln (SM22)	Proteintech	Anti-TAGLN, 10493-1-AP
Acan	Proteintech	Anti-aggrecan, 13880-1-AP
Sox9	Millipore	Anti-Sox9, AB5535
Beta-actin	Sigma-Aldrich	Anti-b-Actin, A5441
